# Atypical Presentation of Lemierre Syndrome Without an Oropharyngeal Source in a Young Adult Male Patient

**DOI:** 10.7759/cureus.95740

**Published:** 2025-10-30

**Authors:** Vaaragie Subramaniam, William Echols, Jessica Houck

**Affiliations:** 1 Emergency Medicine, University of Kentucky, Lexington, USA; 2 Emergency Medicine, University of Kentucky Albert B. Chandler Hospital, Lexington, USA

**Keywords:** antibiotic therapy, fusobacterium necrophorum, internal jugular vein thrombosis, lemierre syndrome, septic thrombophlebitis

## Abstract

A 22-year-old African American male patient presented to the emergency department (ED) with blood-tinged vomiting and chest tightness. Workup in the ED showed acute renal failure with pulmonary septic emboli on computed tomography (CT) imaging. He was admitted to the hospital. During his hospital stay, aerobic/anaerobic venous blood cultures were positive for *Fusobacterium necrophorum, *and an ultrasound of the internal jugular vein revealed a deep vein thrombosis (DVT) consistent with septic thrombophlebitis. He was subsequently diagnosed with Lemierre syndrome. Evaluation for oropharyngeal disease, a more common cause, was negative, making this an atypical presentation. The patient was initially treated with piperacillin-tazobactam, then tailored to metronidazole based on susceptibility for four weeks, and had a full recovery.

## Introduction

Lemierre syndrome is a rare but serious complication of oropharyngeal infections that extends into the lateral pharyngeal spaces, resulting in inflammation and septic thrombophlebitis of the internal jugular vein. This process can lead to septicemia and death [1]. The worldwide incidence is estimated at 1-3.6 cases per 1,000,000 individuals, with a predominance of patients between 19 and 22 years of age [1,2]. Mortality rates have been reported to be as high as 10% [3].

The most common causative pathogen is *Fusobacterium necrophorum*, an obligate anaerobic gram-negative bacillus that often comprises normal oral flora. Typically, this bacterium initiates tonsillitis or pharyngitis before spreading into the pharyngeal spaces; however, these infections can result from odontogenic sources as well. Less commonly, oral *Streptococcus *species have been implicated [2]. The resulting inflammatory response promotes platelet aggregation and disruption of mucosal barriers, leading to the formation of septic thrombi. These thrombi can embolize to distant organs, including the lungs, joints, bones, muscles, and spleen, resulting in significant end-organ damage [4]. In severe cases, septicemia can progress to shock, multiorgan failure, and death, underscoring the importance of early recognition and treatment.

We present a rare case of Lemierre syndrome without evidence of oropharyngeal infection.

## Case presentation

A 22-year-old male patient with no significant past medical history presented to the emergency department (ED) with a chief complaint of vomiting. The patient reported that he had been feeling ill for the previous three days with nasal congestion, fever, myalgias, and vomiting. He was initially seen at an urgent care and diagnosed with influenza and provided oseltamivir. Upon review of symptoms, the patient elicited that he was having blood-tinged emesis. Vitals were only significant for a heart rate of 105 beats per minute (bpm). In the ED, the patient received oral ondansetron and underwent a chest radiograph, which was unremarkable. Laboratory evaluation was not pursued at that time. A Mallory-Weiss tear was suspected due to prolonged vomiting. He was prescribed oral ondansetron and given strict return precautions.

The patient returned to the same ED three days later, due to persistent fever, myalgias, and severe vomiting. He reported no longer being able to tolerate anything by mouth, including his medications. He also reported decreased urine output. Review of systems was positive for constant chest tightness that improved with leaning forward and worsened with lying flat. He stated that hematemesis had resolved and denied hematochezia, diarrhea, headache, dizziness, or shortness of breath. 

Initial vitals in the ED showed he was afebrile with a heart rate of 84 bpm, respiratory rate of 18 breaths per minute, and a blood pressure of 147/90 mmHg. His physical exam was significant for chest tenderness over the sternum that worsened with palpation, epigastric tenderness, and dry mucus membranes. Differential diagnosis included influenza or other viral syndrome, pericarditis, pneumonia, and gastroenteritis.

**Figure 1:**
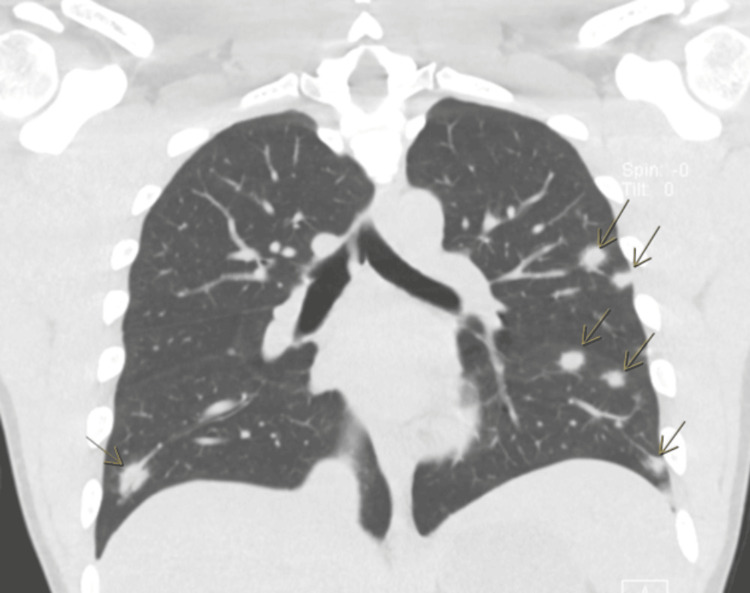
Coronal CT scan of the chest without contrast demonstrating multiple scattered bilateral pulmonary nodules (arrows), consistent with septic emboli. CT: computed tomography

Labs were indicative of sepsis with acute renal failure (Table 1). Blood cultures were obtained, and the patient was treated empirically with 2 g IV cefepime and 1,750 mg IV vancomycin. A computed tomography (CT) scan of the chest, abdomen, and pelvis without contrast was ordered and demonstrated septic emboli with bilateral pulmonary nodules (Figure 1). While in the ED, nephrology, cardiology, and infectious disease were consulted, he was admitted to the internal medicine service with concern for sepsis with multiorgan failure secondary to bacteremia and septic emboli of unknown origin.

**Table 1:** Emergency department laboratory analyses results. WBC: white blood cells; CRP: C-reactive protein; BUN: blood urea nitrogen; ALT: alanine transaminase; AST: aspartate aminotransferase; BNP: b-type natriuretic peptide

Laboratory analysis	Result	Normal reference range
WBC	17.92	3.7-10.3 x 10³/uL
Neutrophils absolute	15.95	1.6-6.10 x 10³/uL
Lymphocytes absolute	0.72	1.2-3.9 x 10³/uL
Monocytes absolute	1.25	0.3-0.9 x 10³/uL
Eosinophils absolute	0.00	0.0-0.5 x 10³/uL
Basophil absolute	0.00	0.0-0.10 x 10³/uL
Echinocytes	Present	-
Target cells	Present	-
Glucose	107	74-99 mg/dL
Sodium	133	136-145 mmol/L
Potassium	3.9	3.6-4.9 mmol/L
Chloride	93	97-107 mmol/L
CO_2_	20	22-29 mmol/L
Creatinine	8.19	0.6-1.1 mg/dL
Anion gap	20	6-16 mmol/L
BUN	72	7-21 mg/dL
Calcium	8.7	8.9-10.2 mg/dL
Albumin	3.1	3.5-5.2 g/dL
Total protein	6.9	6.3-7.9 g/dL
ALT, plasma	23	10-35 U/L
AST, plasma	24	10-35 U/L
Total bilirubin, plasma	1.1	0.2-1.1 mg/dL
Alkaline phosphatase	305	35-104 U/L
Lactate	2.1	0.5-2.2 mmol/L
Procalcitonin	>400.00	<0.09 ng/mL
CRP, plasma	350.9	≤0.8 mg/mL
N-terminal ProBNP, plasma	5360	0-449 pg/mL
Troponin T, high sensitivity, 0 hr	13	<19 ng/L
Troponin T, high sensitivity, 2 hr	13	<19 ng/L

Inpatient course 

While he was admitted to the hospital medicine service, cardiology was consulted due to concerns for pericarditis or myocarditis. They recommended symptomatic management and ordered a transthoracic echocardiogram, which showed no valvular abnormalities or overt findings consistent with endocarditis. Ejection fraction was normal, and there were no findings of a significant pericardial effusion to suggest myocarditis or pericarditis. 

Nephrology was consulted for his acute kidney injury, and they recommended a renal biopsy and hemodialysis due to his kidney function. Interventional radiology placed a tunneled dialysis catheter, and the patient received hemodialysis twice during his hospital stay. The renal biopsy showed his acute kidney injury was due to rapidly progressive glomerulonephritis, and he was started on a trial of prednisone. Infectious disease recommended following blood cultures and testing for chlamydia and gonorrhea, given urinalysis was concerning for a urinary tract infection, which were negative. 

**Figure 2:**
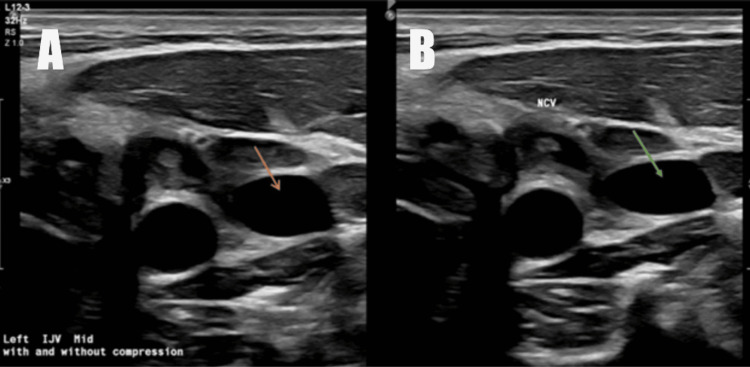
Ultrasound of the left internal jugular vein showing a clot (arrows) without compression (A), which persists with compression (B).

On hospital day 3, the patient’s neck was tender on the left compared to the right. His blood cultures from the first day were positive for *Fusobacterium necrophorum,* so an ultrasound of the neck was ordered. On the ultrasound, the left internal jugular vein had an acute deep vein thrombosis (DVT), which was likely due to septic thrombophlebitis (Figure 2). Since the blood cultures were positive for *Fusobacterium necrophorum* and he had an acute DVT in the left internal jugular vein, the patient was diagnosed with Lemierre syndrome. Infectious disease recommended transitioning the antibiotics to piperacillin-tazobactam 4.5 g IV every 12 hours and linezolid 600 mg IV every 12 hours. Prednisone for his acute kidney injury was stopped due to concern for the spread of his infection. Urine output improved, and the tunneled catheter was removed. 

A panorex was ordered to look for dental disease as the origin of his Lemierre syndrome, but it was negative for the identification of any periapical abscesses. Repeat blood cultures from hospital day 4 were negative, so the piperacillin-tazobactam was discontinued, and he was started on metronidazole 500 mg three times a day for four weeks based on the sensitivities of the bacteria found in the blood cultures from hospital day 1. The patient was discharged after 11 days in the hospital and was advised to follow up with the infectious disease and nephrology outpatient. His kidney function had almost completely recovered (Table 2). In the outpatient setting, the nephrologists determined his kidney function had significantly improved, so no further follow-up was scheduled. 

**Table 2:** Discharge laboratory values demonstrating improvement in renal function and resolution of metabolic abnormalities following treatment for Lemierre syndrome. BUN: blood urea nitrogen

Laboratory analysis	Result	Normal reference range
Blood cultures	No growth at day 5	-
Glucose	101	74-99 mg/dL
Sodium	136	136-145 mmol/L
Potassium	3.9	3.6-4.9 mmol/L
Chloride	97	97-107 mmol/L
CO_2_	26	22-29 mmol/L
Creatinine	3.32	0.6-1.1 mg/dL
Anion gap	13	6-16 mmol/L
BUN	63	7-21 mg/dL
Calcium	9.2	8.9-10.2 mg/dL
Albumin	3.1	3.5-5.2 g/dL

Later that month, the patient developed a sore throat three days after finishing his course of antibiotics. He contacted his infectious disease doctor, who advised him to re-present for further evaluation. In the ED, the patient reported a subjective fever and chills. An ultrasound was done on the patient’s neck, and there was no evidence of persistent internal jugular thrombus. His streptococcal polymerase chain reaction (PCR) was negative, and he was told to return to the ED if his symptoms worsened. He has been doing well since, with complete symptom resolution.

## Discussion

It is unclear how this patient acquired Lemierre syndrome without any evidence of dental or oropharyngeal disease. Infectious disease hypothesized that the vomiting caused by the irritated mucosa of the oropharynx may have led to bacterial translocation, which subsequently progressed to Lemierre syndrome. 

Lemierre syndrome is septic thrombophlebitis of the internal jugular vein. It starts with an oropharyngeal infection, which progresses to inflammation of the internal jugular vein and subsequent bacterial thrombus formation within the vessel. This thrombus can lead to septic emboli, causing sepsis and multiorgan failure. The bacteria responsible for this are typically either *Streptococcus *or *Fusobacterium* species (an anaerobic gram-negative bacillus that constitutes normal flora in the oral pharynx) [2]. 

Assessment for Lemierre syndrome includes blood cultures, CT scan of the chest and neck, and venous duplex ultrasound of the neck to evaluate for thrombus formation [2]. Once the diagnosis is made, treatment involves empiric antibiotics [2]. Options include: piperacillin-tazobactam 3.375g IV every six hours; carbapenem such as imipenem (500 mg IV every six hours) or meropenem (1 g IV every eight hours) or ertapenem (1 g IV every 24 hours); and ceftriaxone (2 g IV every 24 hours) plus metronidazole (500 mg IV every eight hours). If the patient is unstable, vancomycin should be added to cover methicillin-resistant *Staphylococcus aureus*. Antibiotics should be tailored based on susceptibility. Bacteria responsible for Lemierre syndrome (*Fusobacterium necrophorum* and oral streptococci) usually are susceptible to metronidazole, clindamycin, imipenem, amoxicillin-clavulanate, and cefoxitin [2]. Patients are recommended to receive four weeks of antibiotics in addition to two weeks of IV antibiotics and two weeks of oral antibiotics, according to UpToDate [2]. 

Of note, there is some controversy regarding the benefit of anticoagulation use in this patient population [5]. A literature review found that approximately 64% of reported cases were treated with anticoagulation, with the most common choice being low-molecular-weight heparin [5]. The treatment duration of anticoagulation use varied from two weeks to four months [5]. There have been two reported cases of bleeding events associated with anticoagulation use; however, no serious adverse events have been demonstrated [6,7]. No mortality differences were found when comparing anticoagulation versus no anticoagulation; however, larger studies are needed [5].

## Conclusions

Lemierre syndrome is a rare but serious complication of oropharyngeal infections that can lead to life-threatening septic shock and death, particularly when initiation of antibiotic therapy is delayed. The cryptogenic etiology in this patient is unusual, as the disease developed without clear evidence of an oropharyngeal source. Diagnosis typically requires blood cultures, chest CT imaging, and ultrasound evaluation of the internal jugular veins. Standard treatment consists of prolonged antibiotic therapy, generally administered over several weeks. The role of anticoagulation remains uncertain, as current evidence does not clearly demonstrate a morbidity or mortality benefit. Larger studies are needed to better define optimal management strategies for Lemierre syndrome.
